# Music and animal welfare: from behavioral and physiological effects to potential applications in animal husbandry

**DOI:** 10.7717/peerj.20577

**Published:** 2026-06-16

**Authors:** Gang Zhu, Liangkun Wang, Zechen Wu, Lingna Feng, Feng Shao

**Affiliations:** 1 Southwest University, School of Music, Chongqing, China; 2 Southwest University, School of Life Sciences, Key Laboratory of Freshwater Fish Reproduction and Development (Ministry of Education), Chongqing, China

**Keywords:** Music, Animal welfare, Sound perception, Musical behavior

## Abstract

Music, as a complex auditory stimulus, has become a significant topic of interdisciplinary research due to its diverse impacts and underlying mechanisms. Numerous studies demonstrate that music not only profoundly affects human physiological and psychological well-being, but also has important effects on animal behavior and physiology. This review synthesizes previous research and explores the scientific foundations of interactions between music and animals across five key dimensions. Given the heterogeneity of species and interventions, findings were synthesized narratively. First, it explores the neurological basis of animal music perception from a neurobiological perspective, including the evolutionary traits of auditory systems, interspecies differences in music perception, and the neural pathways through which musical stimuli are transmitted within animal nervous systems. Second, using comparative studies across multiple species, it uncovers the distinct ways in which various animals perceive musical elements—such as tempo, rhythm, and pitch—and the behavioral responses they exhibit. Some species even demonstrate rudimentary musical creativity. Third, the review analyzes the positive effects of music on animal neural and endocrine functions, immune function, and welfare, particularly its role in reducing stress responses, improving emotional states, and enhancing quality of life. Finally, this review summarizes recent advances in studies using fish to investigate the effects of music on animals, highlights the broad potential of fish as experimental models in this research field, and provides representative examples illustrating how such findings may contribute to future improvements in animal welfare and the efficiency of aquaculture practices. Moreover, research on music perception in fish may provide indispensable insights into the evolutionary origins and biological foundations of musical perception in vertebrates.

## Introduction

The regulatory effects of music on human physiology and psychology have been extensively supported by empirical research. Once auditory stimuli are encoded by the auditory centers, they activate the functional networks of the prefrontal cortex and the limbic system (Brown, Martinez & Parsons, 2004; Sugihara et al., 2006). Through modulation of autonomic nervous system activity, music can significantly improve heart rate variability (HRV), maintain blood pressure stability, and reduce adrenaline secretion levels (Lieber et al., 2019; Paszkiel, Dobrakowski & Lysiak, 2020; Toader et al., 2023). Multiple clinical intervention studies have also validated the significant efficacy of music therapy in depression alleviation, preoperative anxiety reduction, and pain management (Adiasto et al., 2022; Axelsen et al., 2022; Ettenberger et al., 2021; Gebhart et al., 2020; Lieber et al., 2019). Moreover, the regulatory effects and therapeutic efficacy are influenced by factors such as music type, individual preferences, and acoustic parameters, demonstrating pronounced context-dependent characteristics (Adiasto et al., 2022; Bispham, 2006; Greenberg, Decety & Gordon, 2021; Paszkiel, Dobrakowski & Lysiak, 2020). Furthermore, physiological structures related to human sound perception exhibit certain conservation across non-human animals (Christensen-Dalsgaard et al., 2011; Fritzsch, Schultze & Elliott, 2023; Nikolsky, 2020). These research findings suggest that music, as a structured auditory stimulus, may exert similar effects across different species by activating conserved neural mechanisms related to emotional and physiological regulation.

Recent research has shifted away from a human-centric paradigm, with increasing studies demonstrating that musical stimuli can elicit similar physiological responses and effects across various non-human animals (Bryant, 2013; Filippi et al., 2019; Snowdon, 2021). The acoustic communication systems of certain animals exhibit structural or functional similarities to human music, with some phenomena potentially arising from convergent evolution (Bryant, 2013; Fitch, 2006; Snowdon, 2021). Their acoustic structures (*e.g.*, fundamental frequency harmonics, rhythmic synchrony) can evoke cross-species emotional contagion and coordinated group behaviors (Bryant, 2013; Gray et al., 2001; Lidicker, 2019). In some animal studies, musical interventions can reduce stereotypic behaviors and improve certain stress-related metabolic indicators (such as blood cortisol concentrations and norepinephrine levels) by modulating hypothalamic-pituitary-adrenal (HPA) axis activity (Snowdon, 2021; Zapata-Cardona, Ceballos & Rodriguez, 2024). However, other studies have reported that music’s effects on animal welfare vary according to species, environment, and intervention parameters, potentially resulting in neutral or negative outcomes (Campo, Gil & Dávila, 2005; Kamar & Md Yusof, 2023). These research findings collectively indicate that animals possess the capacity to perceive music and can respond to musical stimuli in ways functionally similar to humans.

However, existing research remains primarily focused on quadrupedal animals, particularly mammals, with limited coverage of other vertebrate groups, documented only in model species such as zebrafish (Barcellos et al., 2018; Dos Santos et al., 2023; Marchetto et al., 2021; Xiao et al., 2025). This raises questions regarding the predictive boundaries of music therapy’s effects across vertebrate taxa. For instance, fish (including bony fish, cartilaginous fish, and lobe-finned fish) represent the most prolific and diverse group within vertebrates, demonstrating exceptional evolutionary plasticity in morphological development and ecological niche occupation (Guinot & Cavin, 2016). Nevertheless, research on music’s effects on fish remains severely limited, which not only constrains our understanding of the cross-species boundaries of music therapy’s efficacy but also fails to fully capitalize on opportunities to utilize music for improving aquaculture productivity and fish welfare. This review summarizes research progress on music’s effects on non-human animal behavior, physiology, and animal welfare, while examining the broad prospects of fish as subjects for music therapy research, with the aim of providing theoretical foundations and practical guidelines for music therapy applications in aquaculture and broader vertebrate welfare domains.

## Survey methodology

### Search strategy

Relevant studies were identified through searches of Web of Science and Google Scholar, with coverage extending to February 2025. In Web of Science, publications were retrieved using the Boolean search strategy TS = (music) AND TS = (animal); the same search terms were applied in Google Scholar. Additional relevant literature was identified by screening the reference lists of key review articles and seminal studies.

### Screening process

The initial literature search retrieved a total of 954 records, the majority of which were not directly relevant to this study. The literature was selectively examined with priority given to peer-reviewed studies on music or structured auditory stimuli in non-human animals that reported clear behavioral, physiological, neural, or welfare-related outcomes, while studies outside this scope or lacking sufficient methodological detail were not emphasized.

### Narrative synthesis

Following the initial literature screening, 137 publications related to music and animals were identified (Table S1). Of these, 55 studies that specifically investigated the effects of music on animals were selected for inclusion in this review. In addition to these 55 studies, supplementary background literature was incorporated, bringing the total number of references cited in this review to 140. Findings from the reviewed literature were synthesized qualitatively using a narrative approach; given the heterogeneity of species and music-based interventions, findings were synthesized narratively and organized into major thematic categories.

### The evolutionary history of animal auditory systems

The evolutionary trajectory of auditory systems illustrates a gradual shift from primitive mechanical sensation to complex sound processing capabilities. In invertebrates, auditory systems had not yet formed distinct organ structures; instead, they relied on mechanoreceptors to detect environmental vibrations. This primitive sensory mechanism laid the foundation for the subsequent evolution of auditory systems in vertebrates (Pfaff, Schultz & Schellhorn, 2019).

**Figure 1:**
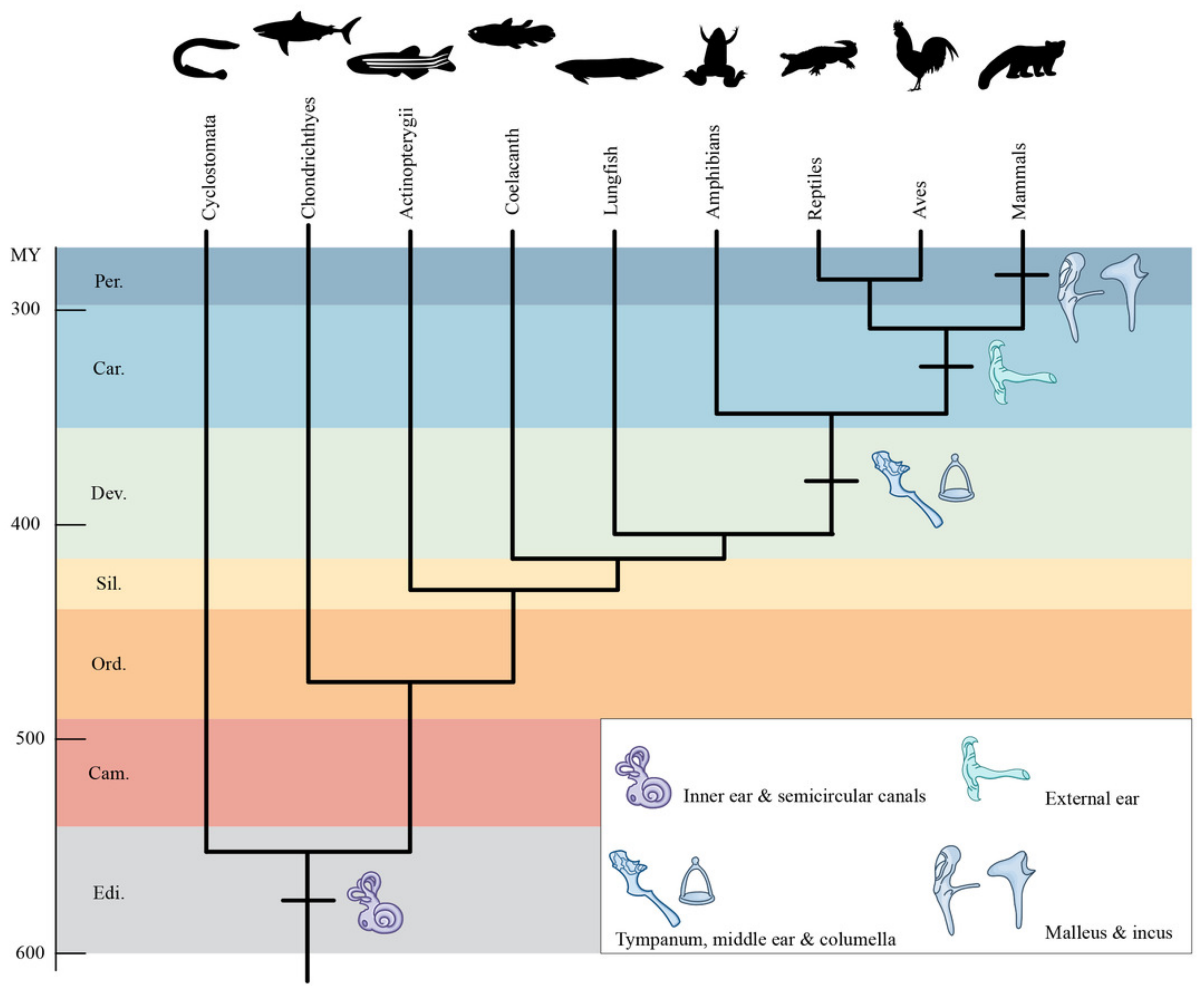
The evolution of the auditory system in vertebrates. Based on the phylogenetic relationships and geological time events provided by the Timetree website (Kumar et al., 2022) , the diagram illustrates the evolutionary trajectory of auditory structures in vertebrates, from cyclostomes to mammals. The earliest auditory structures can be traced back to approximately 600 million years ago in the Precambrian era, when vertebrates evolved the inner ear and semicircular canals, primarily for balance and primitive auditory perception. During the transition of vertebrates from aquatic to terrestrial environments, the auditory system began adapting to airborne sound transmission, leading to the emergence of the tympanum, middle ear, and columella, which facilitated more efficient sound conduction. As reptiles and birds diverged, their auditory systems underwent further specialization. In mammals, the auditory structures reached a highly specialized state with the evolution of three auditory ossicles—the columella (later modified into the stapes), malleus, and incus—which significantly enhanced sound transmission and sensitivity. Overall, the vertebrate auditory system has undergone a progressive evolutionary transition, from simple inner ear structures to the development of middle ear bones, and ultimately to the highly complex auditory ossicles and external ear adaptations. These modifications have enabled different vertebrate groups to meet their auditory demands across diverse ecological environments. Graphics of the organs of the auditory system are original by us, all silhouettes are from PhyloPic (https://www.phylopic.org/), except for one of the silhouettes of *Cetorhinus maximus*, was created by Stephen O’Connor, the silhouette of *Danio rerio*, which was created by Ian Quigley, the silhouette of *Neoceratodus forsteri* was created by Roberto Díaz Sibaja, and the silhouette of *Xenopus laevis*, which was created by Ian Quigley; Both are available under a CC-BY 4.0 licence (https://creativecommons.org/licenses/by/4.0/).

The evolution of vertebrate auditory systems displays distinct stages (Fig. 1). Early vertebrates (such as agnathans and early fish) possessed only inner ear structures, primarily serving vestibular functions (Pfaff, Schultz & Schellhorn, 2019). Although fish are considered to lack structures homologous to those involved in sound reception in tetrapods, fish have developed various organs to perceive sound. For instance, in sharks, hearing is realized by the two displacement systems (cupulae with sensory hair cells) of the lateral line receptors and the labyrinth (Hart & Collin, 2015; Poppelier et al., 2022), whereas in bony fishes several sound transmission systems exist. In the latter, hearing is realized by the close relationship of the swim bladder and ear region or by small gas-filled vesicles extending from the swim bladder (Ladich, 2000; Ladich, 2014; Popper, Hawkins & Sisneros, 2022; Putland, Montgomery & Radford, 2019). A well investigated system for sound transmissions are the Weberian ossicles (tripus, intercalarium, scaphium, and claustrum) of extant and extinct otophysine fishes. A double chain of ossicles connects the inner ear labyrinth with the swim bladder to enhance hearing sensitivity (Bird, Abels & Richardson, 2020; Evans, 1930). An analogous structure to this mechanical sensory system is also found in Chanidae (Actinopterygii, Gonorynchiformes), which presumably enhances audition (Rosen et al., 1970). Furthermore, fish inner ears include a lagena containing a small otolith that contributes to hearing (Baeza-Loya & Raible, 2023; Harada, Kasuga & Tamura, 2001). Research increasingly demonstrates that fish not only utilize these organs to perceive sound but also exhibit capabilities such as sensing particle displacement, detecting sound pressure, and discerning sound frequency and direction (Fritzsch & Elliott, 2023).

As vertebrates transitioned from aquatic to terrestrial environments, their auditory systems underwent significant morphological and functional transformations. Various lineages independently evolved tympanic membranes and middle ear structures, highlighting the importance of sound perception in terrestrial vertebrates (Christensen-Dalsgaard & Carr, 2008; Fettiplace, 2020; Pfaff, Schultz & Schellhorn, 2019; Takechi et al., 2016). Amphibians were the first to develop middle ear structures, marking a significant breakthrough in the auditory systems of land-dwelling vertebrates. Reptiles later developed external ear structures, culminating in mammals with highly integrated inner, middle, and outer ear systems. This evolutionary progression involved not only increased structural complexity but also adaptive molecular changes, including the evolution of auditory-related proteins involved in hair bundle morphology, mechanotransduction, and endolymphatic potential regulation (Fritzsch & Straka, 2014; Lipovsek & Elgoyhen, 2023). These advancements significantly enhanced the ability to detect airborne sound waves, particularly enabling the processing of high-frequency sounds.

However, recent paleontological discoveries reveal that early tetrapods lacked tympanic membranes (Clack, 1989). Some evidence suggests that for as long as 100 million years, early land-dwelling tetrapods might have been unable to hear airborne sounds (Christensen-Dalsgaard & Carr, 2008). The lungfish, the closest living relatives of tetrapods, provide an excellent model for investigating the auditory systems of early tetrapods (Liang, Shen & Zhang, 2013). Christensen and colleagues (Christensen-Dalsgaard et al., 2011; Christensen, Christensen-Dalsgaard & Madsen, 2015) investigated the underwater hearing capabilities of African lungfish in a standing wave tube to observe brainstem and auditory nerve responses. Surprisingly, lungfish were found to detect sound waves at around 200 Hz, and the air within their lungs vibrated along with the sound waves. Computed tomography (CT) imaging confirmed that lungfish could detect sound pressure through resonant vibrations of their pulmonary cavities and perceive airborne sounds *via* head vibrations. This indicates that tetrapod ancestors may have possessed pressure-based hearing mechanisms, capable of underwater pressure detection and rudimentary aerial hearing before transitioning to land (Capshaw, Christensen-Dalsgaard & Carr, 2022; Fritzsch & Elliott, 2023). These sensory systems provide the fundamental physiological conditions for fish to perceive musical elements such as rhythm and frequency. Studies on lungfish demonstrate that they can perceive airborne sounds to some extent through resonance of the head and lung cavity, suggesting that tetrapod ancestors or even earlier fish may have already possessed the ability to perceive airborne sounds. However, current research on airborne sound perception in fish is rarely reported, which highlights the necessity and potential value of conducting sound and music perception studies in fish.

### Bioacoustic properties of sound in animal habitats

Understanding acoustic properties is crucial for explaining how animals interact with their acoustic environment. Key acoustic features include amplitude, frequency, and wavelength. Amplitude, measured in decibels (dB), quantifies sound pressure levels, while frequency, measured in Hertz (Hz), reflects the rate of sound wave oscillations and correlates positively with pitch (Kinsler et al., 2000; Plack, 2023). The soundscape is comprised of two primary components: musical tones and noise. Musical tones exhibit periodic vibrations with stable frequencies and waveforms (*e.g.*, the fundamental frequencies of piano or violin sounds), whereas noise consists of non-periodic vibrations lacking a fixed frequency. In contemporary music, noise is often used as a creative element (*e.g.*, the acoustic characteristics of cymbals or snare drums) (Kinsler et al., 2000).

Animals’ ability to perceive acoustic signals varies significantly among species. Humans, for example, have a hearing range of 20–20,000 Hz and are most sensitive to the 1,000–4,000 Hz range. Some species, however, can detect infrasound (<20 Hz) or ultrasound (>20,000 Hz), and certain animals possess dual sensitivity to both (Hewitt, 2008; Moore, 2012; Plack, 2023). Studies have indicated that sound pressure levels exceeding 85 dB can markedly affect animal behavior and physiological states (Kinsler et al., 2000; Moore, 2012).

Natural acoustic environments consist of diverse sound sources, including geological activities (such as wind, precipitation, and earthquakes), biological activities (such as animal communication), and human activities (such as transportation and industry) (Erbe et al., 2022). Background noise can reduce signal-to-noise ratios (SNR), interfering with animals’ ability to detect and interpret communication signals. This, in turn, can influence social behavior and ecological adaptability. Such impacts vary across temporal and spatial scales. Short-term disturbances (like brief noise events) typically lead to transient behavioral adjustments (*e.g.*, changes in activity patterns), whereas long-term habitat fragmentation or prolonged noise pollution may cause lasting shifts in habitat use patterns. For instance, urbanization-induced noise pollution has been shown to alter hedgehogs’ spatial distributions and activity rhythms (Berger et al., 2020). In another example, sudden spikes in sound pressure levels during zoo concerts have been observed to trigger immediate changes in over 20 different animal behavior patterns (such as activity rhythms and stress responses), suggesting that such events might pose potential risks to animal welfare (Harley et al., 2022). In agricultural settings, the sound environment, including mechanical noise, animal vocalizations, and music interventions, plays a significant role in modulating animals’ behavioral patterns and psychological states (Ginovart-Panisello et al., 2020). To enable precise sound environment management, researchers have developed deep learning-based animal sound recognition systems. Combined with real-time noise filtering technology, these systems offer intelligent solutions for dynamically monitoring animal health conditions (Jung et al., 2021).

The influence of the acoustic environment is particularly pronounced in aquatic environments. For example, comparative evolutionary studies indicate that humpback whales sing in water using melodies and thematic variations resembling human music. Their songs exhibit rhythmic patterns, intervals, repeated sections, and an ABA structure of statement, development, and recapitulation (Arnon et al., 2025). Studies on Cyprinidae have demonstrated that acoustic signals are critical to reproductive behaviors, especially in courtship and pair bonding. These sounds not only serve as a means of communication but also have profound effects on interspecies competition and population structure (Wang et al., 2022). In terms of cognitive ability, benthic sharks have been shown to use auditory cues for associative learning, although they exhibit some limitations in recognizing complex sounds (Vila Pouca & Brown, 2018). This suggests that fish auditory systems may possess specialized functions developed through evolutionary processes. Moreover, the impact of anthropogenic noise has been well documented. Even moderate levels of anthropogenic noise can significantly affect the behavior and cognition of species like the ambon damselfish, disrupting their foraging and social behaviors (De Souza et al., 2022). These findings highlight the need to address noise pollution’s potential risks alongside traditional conservation priorities, such as species numbers and habitat protection.

It is worth noting that music, as a highly structured and emotionally expressive form of sound, differs from conventional noise and environmental sounds. Its rhythm, melody, and harmony have unique physiological and psychological effects on animals. For example, rats can perceive rhythmic changes in music and exhibit more pronounced nose-poking responses when faced with unfamiliar rhythms (Celma-Miralles & Toro, 2020). This suggests that music may serve as an effective tool for improving animal welfare and regulating their emotions.

### The influence of music on animal behavior

Studies have shown that natural sounds—such as wind, flowing water, and birdsong—can significantly influence human emotions and behavioral responses (Stomberg & Tiderman-Österberg, 2021). While research on the effects of natural sounds on non-human animals is rarely reported, studies typically focus on how music or artificial sounds affect animals’ behavioral and emotional responses (Berger et al., 2020; Erbe et al., 2022; Zapata-Cardona, Ceballos & Rodriguez, 2024). This research trend has also prompted exploration into the potential effects of music on animals. Different species exhibit specific behavioral and physiological responses under musical environments. Overall, the promotive effects of music on learning and cognitive functions have been most systematically and robustly studied in model mammals such as laboratory rats (Kühlmann et al., 2018). For example, rats exposed to music during their juvenile stages demonstrate faster extinction of conditioned fear responses and reduced anxiety levels in adulthood (Chen et al., 2019). Regular music listening has also been confirmed to improve rats’ performance in spatial learning tasks (Korsós et al., 2018). Research has further revealed that broiler chickens receiving sound stimulation during the embryonic period exhibit lower stress responses in adulthood, suggesting that music or rhythmic sounds may also possess the potential to regulate emotional and neural functions in poultry (Ahmad-Hanafi et al., 2024). This phenomenon has been observed across multiple species. For example, live musical performances have been shown to significantly affect the activity levels and social behaviors of Fiordland penguins (Fanning, Larsen & Taylor, 2020), while pigs exposed to different types of music exhibit significant changes in anxiety levels and social interactions (Li et al., 2019). These studies suggest that music, as an environmental stimulus, may produce observable regulatory effects on animal behavior under specific conditions, though its effects typically demonstrate high species-specificity and context-dependency.

Among the specific characteristics of music, elements like pitch, rhythm, and tempo have a particularly pronounced impact on animal behavior. Research shows that low-frequency rhythms help relax dogs and reduce anxiety, whereas high-frequency rhythms may increase their activity levels (Amaya et al., 2020a). This pattern is also evident in other species: classical music has been found to significantly alleviate stress responses in horses (Huo et al., 2021) and enhance prosocial behaviors in bottlenose dolphins (Guérineau et al., 2022). Captive western lowland gorillas show activity and social behavioral changes depending on musical tempo and pitch, with low-tempo music reducing anxiety-related behaviors (Brooker, 2016). Similarly, auditory stimulation with classical music has been shown to reduce stereotypic behaviors in zoo-housed Asian elephants (Wells & Irwin, 2008). Further studies reveal the underlying mechanisms of these phenomena, suggesting that animals can analyze different sound parameters, such as frequency, rhythm, and timbre, to perceive and distinguish musical cues, thereby adjusting their behaviors accordingly (Fan et al., 2022).

In veterinary clinic settings, music has proven to significantly improve animal welfare by alleviating stress responses. Research indicates that classical music can effectively reduce anxiety and fear, improving behavioral reactions during veterinary examinations or treatments (Riemer et al., 2021). In clinical environments, animals often experience negative emotions such as anxiety and fear due to unfamiliar surroundings and medical procedures, which not only impact their welfare but can also pose safety risks for veterinary staff. Consequently, music therapy offers a non-invasive intervention strategy that can improve behavioral responses, reduce stress, and enhance the overall veterinary experience for animals (Bergh et al., 2021).

However, caution is necessary when using music as an enrichment tool in artificial environments. Researchers emphasize that musical stimuli must be carefully designed to align with animals’ natural behaviors and adaptive capacities, avoiding overstimulation or discomfort (Piitulainen & Hirskyj-Douglas, 2020). Prolonged exposure to inappropriate acoustic environments can lead to stress responses in animals, thereby altering their physiological and behavioral patterns, a concern that is especially pronounced in urban settings (Erbe et al., 2022). Such effects are even more pronounced in wildlife. Although some species, such as hedgehogs, can adapt to temporary environmental disturbances, persistent changes in their habitat may result in reduced activity ranges and lower reproductive success rates (Berger et al., 2020).

### The influence of music on animal neural and endocrine functions

Animal behavior is closely tied to neural activity, and examining the impact of musical environments on endocrine function offers a deeper understanding of how music influences animal behavior. From a neurobiological perspective, existing research has demonstrated that music can influence animal emotion, reward responses, and stress regulation by modulating neurotransmitter systems including dopamine, serotonin, and norepinephrine (Dos Santos Lemes Lechuga et al., 2023; Lee et al., 2017; Panksepp, 1986). Neurobehavioral evidence shows innate rhythmic perception abilities across species—from rodents to cetaceans—manifesting as phase-locking responses of the midbrain dopaminergic system to isochronous beats (Clack, 1989; Lidicker, 2019). Research has demonstrated that music can regulate dopamine secretion dynamics related to reward and pain perception in humans (Ferreri et al., 2019). However, findings from mouse studies indicate that music’s effects on dopaminergic dynamics may vary by gender, suggesting that auditory responses exhibit gender-dependent characteristics (Flores-García et al., 2025). This emphasizes the importance of considering gender differences when conducting music therapy. As a key neurotransmitter, dopamine not only regulates emotional and reward systems but is also intimately involved in the generation and maintenance of behavioral motivation, providing crucial insights into the neurobiological basis of music therapy (Chanda & Levitin, 2013).

In terms of physiological adaptability, music effectively modulates stress responses. Studies have shown that prolonged exposure to music can enhance horses’ tolerance to sudden environmental stimuli and improve their ability to adapt, especially in response to acute stress events (Huo et al., 2021). The regulatory effects of music also extend to physiological indicators, such as improved hypothalamic-pituitary-adrenal axis function. Long-term observations reveal that music can modulate autonomic nervous system activity, enhancing heart rate variability. This underscores music’s non-invasive and effective role in managing stress responses and promoting physiological stability (Amaya et al., 2020b).

Music also plays a critical role in neural development. Research has demonstrated that appropriate musical stimulation during intrauterine or infant stages can promote cognitive and memory neural activities, exerting positive effects on nervous system development (Poćwierz-Marciniak & Harciarek, 2021). Compared to groups that did not receive music exposure, prenatal and lactational music listening can slow the decline rate of Brain-Derived Neurotrophic Factor (BDNF) levels in piglets from birth to weaning, suggesting that musical stimulation may have potential effects on animal nervous systems (Lippi et al., 2022). Such early interventions not only affect immediate behavioral performance but may also have lasting effects through epigenetic regulation. Further studies have highlighted the importance of this neuroplasticity in forming social bonds, particularly during the establishment of mother-offspring relationships (Cainelli, Vedovelli & Bisiacchi, 2024). Appropriate sound stimulation fosters neural development and strengthens emotional connections, which has practical implications for livestock rearing and management (Chatterjee, Hegde & Thaut, 2021; Orihuela et al., 2021).

Zebrafish models offer deeper insights into these processes. Studies have shown that solfeggio frequency music can reverse cognitive deficits and abnormal cortisol levels caused by 24-hour light exposure (Dos Santos et al., 2023). This finding not only confirms music’s role in regulating the neuroendocrine system but also suggests that this mechanism might be conserved across species. In the context of environmental enrichment, moderate exposure to classical music has been found to enhance zebrafish welfare (Barcellos et al., 2018), while auditory enrichment environments (AEE) can alleviate anxiety-like behaviors induced by social isolation (Marchetto et al., 2021). These findings indicate that music not only regulates individual emotional and behavioral responses but also has far-reaching implications for animal social behavior and adaptability.

However, research has also revealed a dual nature to music’s effects. In instances of catechol-induced autism-like behaviors, music unexpectedly exacerbated negative outcomes, challenging the general assumption of music therapy’s universal benefits (Xiao et al., 2025). The researchers speculated that oxidative stress imbalance was a potential cause of abnormal behavior in zebrafish, suggesting that we need to consider the complexity of cross-species research when conducting music therapy experiments.

### The influence of music on animal immune function

As a unique form of acoustic stimulation, music can influence animals’ physiological and biochemical responses through various pathways, notably impacting their immune system. Studies have shown that musical stimuli not only significantly alter animal behavior patterns but also affect cortisol levels and immune functions, particularly humoral immunity. These effects are closely tied to the type of music, the intensity of the stimulation, and the environmental conditions, suggesting that music, as an environmental factor, modulates animals’ immune systems *via* the hypothalamic-pituitary-adrenal axis (Li et al., 2021a).

In livestock, the impact of music on immune function is especially pronounced. Research indicates that classical music played under different stocking densities can effectively reduce stress levels in broiler chickens, significantly enhancing their antioxidant capacity and immune function. These immunomodulatory effects are reflected not only in improved physiological indicators but also in better meat quality, demonstrating that music can improve overall health by mitigating stress responses and thus promoting productivity. Specifically, music interventions reduce the release of stress hormones and alleviate oxidative stress, which would otherwise negatively affect the immune system, thereby strengthening immune responses (Gao et al., 2023). Studies on pregnant animals further highlight the immune-regulating effects of music. For example, appropriate musical stimulation improves the physiological state of pregnant sows and reduces stress responses. This improvement benefits the mother’s immune function and may also influence fetal immune system development through the maternal-fetal interface, indicating that music interventions support maternal health and indirectly promote fetal immune development (Silva et al., 2017).

Early musical interventions also hold unique significance for immune system development in young animals. Research suggests that early exposure to music can enhance piglet neural development, ultimately improving their overall well-being. This optimized neural development may positively impact the immune system’s growth and maturation *via* the interplay of the neuro-endocrine-immune network. In particular, music stimulation may enhance neuroplasticity, further influencing the development and function of immune cells and thereby improving immune responses (Lippi et al., 2022). In large livestock animals, music has also been shown to significantly affect immune function. Carefully managed musical stimulation improves dairy cattle behavior and has a positive impact on leukocyte populations. This suggests that music not only indirectly enhances immune function by reducing stress but also directly influences immune cell regulation, particularly in cytokine secretion, thereby promoting immune cell proliferation and differentiation (Contreras-Torres et al., 2024).

However, the effects of music on animal immune function are bidirectional. While appropriate musical stimulation can enhance immunity, excessive or inappropriate music can act as a stressor, suppressing immune function. Studies have shown that selecting the right parameters—such as frequency, rhythm, and volume—is critical for immune regulation. Therefore, it is essential to tailor music interventions to the species, physiological stage, and environmental conditions of the animals in question (Kühlmann et al., 2018).

### The impact of music on animal welfare

As a tool for environmental management, music plays a crucial role in enhancing the welfare of animals housed in artificial environments. Music interventions are increasingly applied to enhance animal welfare and optimize production practices (Zapata-Cardona, Ceballos & Rodriguez, 2024). As a core strategy of environmental enrichment, music modulates the activity of the hypothalamic-pituitary-adrenal axis (HPA axis), reducing stereotypic behaviors and abnormal metabolic indicators in captive animals (Snowdon, 2021; Zapata-Cardona, Ceballos & Rodriguez, 2024). In livestock scenarios, slow music interventions (60-80 bpm) have been shown to increase daily milk yield in high-yielding buffalo by 12.7% and lower subclinical mastitis indicators such as somatic cell count (SCC) in milk (Abuzead & Khalil, 2007). Innovative technologies such as music enrichment strategies informed by social network analysis (Chase, 2006) are driving precision management of animal welfare. These findings provide a theoretical framework and practical paradigm for establishing welfare assessment systems based on bioacoustic markers (*e.g.*, salivary cortisol, HRV spectra) and for developing species-specific music enrichment protocols (Zapata-Cardona, Ceballos & Rodriguez, 2024).

Research shows that playing classical music in animal shelters improves dogs’ behavior and health conditions (Amaya et al., 2020a; Cyroń, Czyz & Patkowska-Sokoła, 2018; Lindig, McGreevy & Crean, 2020), whereas environmental noise exacerbates stress responses in cats, harming their welfare (Eagan, 2008). As a non-invasive intervention, appropriately chosen music can promote emotional stability, effectively reducing animals’ anxiety and fear during veterinary examinations and treatments (Riemer et al., 2021). For dogs, slow-tempo music has been found not only to significantly alleviate anxiety but also to lower heart rates (Engler & Bain, 2017; Georgiou et al., 2024). Similar positive effects are observed in cats, especially in stress-inducing environments like veterinary clinics (Hampton et al., 2020). However, the limitations of music intervention should not be overlooked. Long-term exposure to inappropriate acoustic environments, particularly in urban settings, may trigger stress responses, negatively impacting physiological and behavioral patterns (Erbe et al., 2022). Therefore, carefully selecting the type of music and frequency of playback is essential for ensuring the effectiveness of such interventions.

In modern animal husbandry, music as an enrichment tool has demonstrated unique value in improving production performance indicators. In poultry farming, for example, music-based enrichment strategies have received empirical support. Studies reveal that combining soothing music with controlled strobe lighting can significantly enhance broiler chickens’ autonomous behaviors (such as exploratory activities and group interactions), increase feeding frequency, and reduce plasma cortisol levels, indicating effectively mitigated stress responses (Jacob et al., 2022). These findings suggest that music can modulate physiological states and behavioral patterns, enhancing overall welfare, which is especially valuable in intensive farming systems (Laurijs et al., 2021).

The welfare benefits of music for sows are equally noteworthy. Research shows that musical stimulation during gestation can significantly lower salivary cortisol concentrations in sows and reduce the occurrence of stereotypic behaviors (Silva et al., 2017). When combined with positive human-animal interactions (*e.g.*, gentle tactile stimulation), music further enhances emotional stability and reproductive performance. Studies report a 12.3% increase in litter size under such interventions (Hayes et al., 2021). This multimodal approach not only improves emotional well-being but also yields tangible benefits for productivity.

In dairy production systems, music interventions have also achieved significant results. Playing classical music at andante tempo (60-80 BPM) during milking sessions can increase milk yield by 6.8% and reduce somatic cell counts, reflecting improved udder health (Kochewad et al., 2021). Certain musical parameters such as slow tempo music, significantly reduce buffalo milk cortisol levels (*P* < 0.05), shorten the latency of stress-induced behaviors (Abuzead & Khalil, 2007), and extend the duration of rumination in cattle (Snowdon, 2021). Furthermore, music stimulation can enhance oxytocin release *via* hypothalamic-pituitary axis activation, boosting milking efficiency (reducing milking time by 18%) and decreasing defensive behaviors like kicking (Kemp, 2020). These outcomes provide a solid physiological foundation for the implementation of music interventions in animal husbandry.

### Fish as emerging models in music-related animal research

Compared to other vertebrates, fish have long been relatively underrepresented in music therapy-related research. However, their unique sensory biology and widespread application in aquaculture practices have gradually established fish as important research subjects for investigating the effects of music on animals. A growing body of evidence demonstrates that fish possess the capacity to perceive musical stimuli, and this perceptual capability can further influence multiple aspects of their biology, including growth performance, behavioral responses, and physiological states (Singh & Sharma, 2022; Toth, Bársony & Kusza, 2025). For instance, goldfish can distinguish music from other types of sounds (Imanpoor, Gholampour & Zolfaghari, 2011), and are further capable of discriminating between different musical genres (Shinozuka, Ono & Watanabe, 2013). Similarly, carp and sharks have demonstrated the ability to discriminate between different musical genres, such as classical music and jazz music (Chase, 2001; Vila Pouca & Brown, 2018). These findings provide an important empirical foundation for understanding the mechanisms underlying the effects of music on fish.

Current research on music therapy in fish has primarily focused on its effects on aquaculture-related production parameters. Multiple studies have demonstrated that musical stimuli, particularly classical music, can moderately improve growth performance in fish and influence various production-related metrics, including feed conversion ratio (FCR) and fatty acid composition. For instance, exposure to Mozart’s music has been shown to significantly enhance growth performance and feed conversion efficiency in carp (Papoutsoglou et al., 2010), while concurrently reducing stress response levels (Papoutsoglou et al., 2007). In gilthead sea bream, Mozart’s music promotes maximum growth rate, body weight gain, and improved feed conversion ratio by modulating brain neurotransmitter levels, thereby maintaining individuals in a relatively relaxed state (Papoutsoglou et al., 2008; Papoutsoglou et al., 2015). Additionally, slow-tempo music has also been reported to promote visceral fat accumulation in turbot (Catli, Yildirim & Turker, 2015). For rainbow trout, musical stimulation similarly resulted in significantly improved growth efficiency compared to white noise treatment or control groups (Papoutsoglou et al., 2013). Beyond growth performance, musical stimulation may also alleviate stress experienced by ornamental fish in aquaria lacking environmental enrichment through reducing levels of cortisol and other stress-related hormones, thereby exerting positive effects on animal welfare improvement (Yang et al., 2025; Niu et al., 2025).

In zebrafish, a model organism, research on music therapy has primarily focused on its effects on neural and endocrine regulatory processes. Studies have demonstrated that auditory enrichment can alleviate anxiety-like behaviors exhibited by zebrafish following 24-hour acute social isolation (Marchetto et al., 2021). Zebrafish exposed to classical music for 15 consecutive days exhibited significantly reduced anxiety levels in the novel tank test, accompanied by decreased peripheral pro-inflammatory cytokine levels and enhanced expression of certain central nervous system-related genes (Barcellos et al., 2018). Further studies revealed that chronic exposure to Solfeggio-frequency music, used for meditation, reduced cortisol levels in zebrafish, thereby exerting positive modulatory effects on their cognitive function and endocrine responses (Dos Santos et al., 2023).

It should be noted that the effects of music on fish are also bidirectional. When music intensity exceeds optimal levels, it may be perceived by fish as noise, thereby exerting negative effects. Under laboratory conditions, studies have found that musical stimulation can exacerbate behavioral abnormalities in zebrafish induced by catechol, accompanied by elevated oxidative stress levels (Xiao et al., 2025). Additionally, high-intensity music, as a form of anthropogenic noise, has been reported to increase anxiety-like behaviors and impair memory performance in damselfish (De Souza et al., 2022). Beyond laboratory settings, continuous music noise in natural environments can similarly exert adverse effects on organisms. For instance, noise pollution from coastal music festivals has been found to elevate stress levels in marine species such as the Gulf toadfish (*Opsanus beta*) (Cartolano et al., 2020).

**Figure 2:**
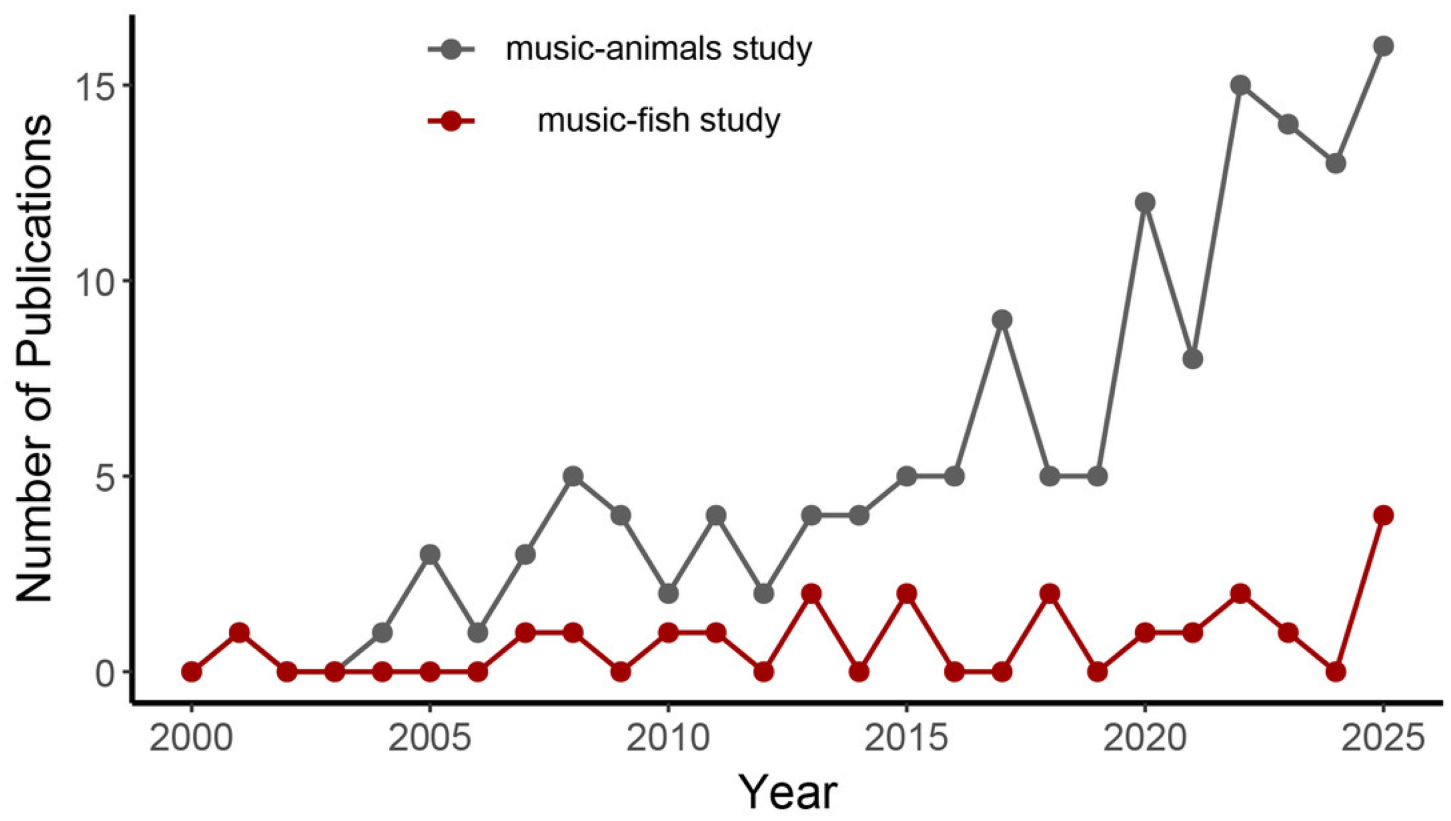
Trends in the number of publications on the effects of music on animals and fish (2000–2025). The grey line represents the annual number of studies examining the effects of music on animals, while the red line indicates studies specifically focusing on fish. Overall, the number of publications on music–animal interactions has increased steadily over the past two decades, reflecting growing research interest in this field. In contrast, studies addressing the effects of music on fish remain relatively limited, although a gradual increase has been observed in recent years. This trend highlights both the emerging attention to fish in music-related animal research and the potential for further exploration in this area. The publication counts were compiled from studies published between January 2000 and December 2025.

The studies outlined in this article focus primarily on the effects of music on tetrapods, with research on fish being relatively scarce. In recent years, significant progress has been made in understanding how music affects animals, though the underlying mechanisms and principles remain to be fully elucidated. Fish, given their ecological importance and unique auditory systems adapted to aquatic environments, present a promising avenue for research on how music influences animal welfare. While research on other animals is relatively abundant, the foundation for studies on fish and music has also gained attention (Fig. 2). A growing body of literature on sound wave transmission and auditory responses in fish provides a theoretical framework and experimental basis for understanding how music might impact their behavior and physiology.

### Evolutionary origins of auditory perception and emotional regulation in fishes

From an evolutionary and developmental biology perspective, lungfish offer a unique lens through which to study emotional regulation in fish. The West African lungfish (*Protopterus annectens*), a species of significant biological interest, underwent complex adaptive changes during its transition from aquatic to terrestrial environments. The water-to-land transition of vertebrates required the evolution of a series of body innovations (Ashley-Ross et al., 2013), the respiratory, sensory, locomotory, circulatory, and other systems had to be remodeled for terrestrial adaptation (Long & Gordon, 2004). Specifically, the compartmentalization and connectivity of the limbic system (particularly the amygdala) related to emotional regulation were significantly enhanced (Bruce & Neary, 1995). Researchers have found that lungfish have already developed the basic structural foundation of the amygdala (González & Northcutt, 2009), suggesting that lungfish possess neural structural foundations similar to terrestrial vertebrates in their emotional regulatory systems.

Genomic studies of lungfish reveal that during the aquatic-to-terrestrial evolutionary transition, their emotion-related gene systems had already been preliminarily established and conservatively maintained. These include neuropeptide S (Nps) and its receptor Npsr1, which are considered to be associated with arousal states and anti-anxiety responses. Additionally, two GABA pathway-related genes, IgSF9b and Arfgef1, exhibit unique coding region variations that participate in maintaining inhibitory synaptic structures within the amygdala and regulating GABA receptor function, respectively. These results suggest that core molecular mechanisms regulating emotional states such as anxiety may have already existed in the common ancestor of lungfish and tetrapods (Wang et al., 2021). Comparative genomic studies across three lungfish species—African, South American, and Australian lungfish—demonstrate that these genes play a central role in emotional regulation and have remained conserved throughout evolution (Schartl et al., 2024; Zhang et al., 2023). These changes provide valuable insights into the emotional regulatory mechanisms in fish. The neural core modules regulating anxiety and fear already existed in aquatic ancestors, indicating that emotional regulation is not a neural function unique to tetrapods.

The genetic conservation of these pathways offers potential clues about how music might affect fish emotions. Fish may rely on these conserved genes to regulate emotional responses, particularly in stressful environments. Music, as an environmental stimulus, might influence fish emotional states through these molecular pathways. For instance, the anti-anxiety genes in lungfish may have played a significant role in their environmental adaptations. This mechanism could also apply to how music modulates fish emotions, opening new directions for research into how music can be used as an environmental intervention to regulate fish emotions. More specifically, music might help fish cope with negative stimuli in their environments by activating genes and neural circuits associated with anxiety regulation. This potential mechanism may be present in many fish species. Lungfish studies thus not only provide critical insights into the genetic basis of emotional regulation in fish but also offer theoretical support for future applications of music interventions to improve fish welfare, particularly in aquaculture and laboratory animal welfare contexts.

As previously mentioned, a critical question in the evolution of terrestrial vertebrates is how they developed the ability to perceive sound. Studies on African lungfish suggest that the aquatic ancestors of tetrapods may have been pre-equipped for aerial hearing through a high frequency extension of their sensory epithelia, driven by pressure hearing *via* air breathing in water (Christensen, Christensen-Dalsgaard & Madsen, 2015). Both lobe-finned fish (such as lungfish) and extant ray-finned and cartilaginous fishes have evolved unique physiological mechanisms to detect sound vibrations. By modifying their sensory organs, different fish species have developed diverse mechanisms to perceive sound. These adaptations not only highlight the complexity of sound processing in fish but also provide new perspectives on how auditory stimuli, including music, might influence their emotional states.

### Zebrafish as a model system for studying music-induced responses

As a transparent model organism, zebrafish offer unique research advantages. Zebrafish share around 70% of their genome with humans, making them highly comparable for studies in human biology (Howe et al., 2013). Zebrafish possess well-developed physiological and anatomical structures for perceiving sound, which makes them ideal candidates for auditory research. Zebrafish possess inner ears and semicircular canals, with lateral line hair cells that exhibit similar structure and function to mammalian cochlear hair cells (Lukasz & Kindt, 2018). They also possess neurotransmitters such as dopamine and stress neuroendocrine axes (Barcellos et al., 2018).

Zebrafish larvae are transparent, facilitating optical imaging. Researchers can utilize transgenic expression of calcium indicators to perform whole-brain calcium imaging at single-cell resolution, recording and analyzing neural activity induced by auditory stimuli (Constantin et al., 2020; Hasani et al., 2023). Beyond optical imaging, auditory function in zebrafish can be measured through electrophysiological methods such as auditory evoked potentials (AEP) (Wong et al., 2022), which will help quantify the effects of music on the auditory system in future studies. Furthermore, optogenetic techniques enable precise manipulation of auditory receptors or central neurons, allowing direct investigation of the relationship between auditory signal encoding and behavioral output (Monesson-Olson et al., 2014).

Music produces diverse effects on animal emotions (Snowdon, 2021; Zapata-Cardona, Ceballos & Rodriguez, 2024). Zebrafish have established mature behavioral measurement systems, such as the Novel Tank Test (NTT) and Light/Dark Test (Wong et al., 2022). Multiple independent studies demonstrate that anxiety-like behaviors in zebrafish are sensitive to sound, and the obtained behavioral indicators possess good statistical power (Barcellos et al., 2018; Johnson et al., 2023; Lara & Vasconcelos, 2021). These methods can be utilized to quantitatively assess the effects of music therapy on anxiety levels. Zebrafish offer abundant genetic research tools, enabling CRISPR/Cas9-mediated precise knockouts, TALENs gene knock-ins, and various transgenic fluorescent markers (Li et al., 2021b).

In other words, zebrafish can serve as an “all-in-one” research platform—achieving music intervention, high-throughput behavioral phenotype screening, *in vivo* neural imaging and electrophysiological mechanism exploration, as well as functional validation based on gene editing or pharmacological intervention within the same model, thereby completing the entire research pipeline from phenotype discovery to mechanism elucidation to mechanism validation.

## Conclusions

Music has increasingly been recognized as a promising environmental enrichment strategy capable of improving animal welfare by modulating behavioral, physiological, and emotional responses. Evidence from studies on mammals, birds, and fish indicates that auditory stimulation can influence stress regulation and neuroendocrine activity, highlighting the potential of music as a low-cost, non-pharmaceutical management tool in agricultural and laboratory settings. Notably, fish represent an important yet underexplored group in this field. Many fish species possess well-developed auditory systems and conserved neuroendocrine pathways involved in stress and emotional regulation, suggesting that they may respond to acoustic stimuli in ways comparable to other vertebrates. Expanding research on fish will therefore not only advance understanding of music’s biological effects in aquatic species but also provide valuable insights into the evolutionary origins of sound perception and emotional regulation across vertebrates. Future interdisciplinary studies integrating behavioral, neurophysiological, and genomic approaches will be essential for elucidating the mechanisms underlying music-induced responses and for developing evidence-based strategies to improve animal welfare in aquaculture and other management systems. Such research will also highlight the value of fish as experimental models for studying the biological effects of music and provide insights into the evolutionary origins of acoustic perception and emotion-related responses in vertebrates.

## Supplemental Information

10.7717/peerj.20577/supp-1 Supplemental Information 1 List of 130 included publications From the 1,854 articles retrieved from Web of Science, 130 articles were identified as reporting the effects of music on animals.

## Additional Information and Declarations

### Data Availability

The following information was supplied regarding data availability: Raw data was not generated in this literature review.

## References

[ref-1] Abuzead SMM, Khalil AM (2007). Behavioral and physiological influences of listening slow and fast music on milking buffaloes. Assiut Veterinary Medical Journal.

[ref-2] Adiasto K, Beckers DGJ, Van Hooff MLM, Roelofs K, Geurts SAE (2022). Music listening and stress recovery in healthy individuals: a systematic review with meta-analysis of experimental studies. PLOS ONE.

[ref-3] Ahmad-Hanafi S, Zulkifli I, Ramiah SK, Chung ELT, Kamil R, Sazili AQ, Mashitah J (2024). Prenatal auditory stimulation and impacts on physiological response to feed restriction in broiler chickens at market age. Poultry Science.

[ref-4] Amaya V, Descovich K, Paterson MBA, Phillips CJC (2020a). Effects of music pitch and tempo on the behaviour of kennelled dogs. Animals.

[ref-5] Amaya V, Paterson MBA, Descovich K, Phillips CJC (2020b). Effects of olfactory and auditory enrichment on heart rate variability in shelter Dogs. Animals.

[ref-6] Arnon I, Kirby S, Allen JA, Garrigue C, Carroll EL, Garland EC (2025). Whale song shows language-like statistical structure. Science.

[ref-7] Ashley-Ross MA, Hsieh ST, Gibb AC, Blob RW (2013). Vertebrate land invasions-past, present, and future: an introduction to the symposium. Integrative and Comparative Biology.

[ref-8] Axelsen JL, Meline JSJ, Staiano W, Kirk U (2022). Mindfulness and music interventions in the workplace: assessment of sustained attention and working memory using a crowdsourcing approach. BMC Psychology.

[ref-9] Baeza-Loya S, Raible DW (2023). Vestibular physiology and function in zebrafish. Frontiers in Cell and Developmental Biology.

[ref-10] Barcellos HHA, Koakoski G, Chaulet F, Kirsten KS, Kreutz LC, Kalueff AV, Barcellos LJG (2018). The effects of auditory enrichment on zebrafish behavior and physiology. PeerJ.

[ref-11] Berger A, Barthel LMF, Rast W, Hofer H, Gras P (2020). Urban hedgehog behavioural responses to temporary habitat disturbance *versus* permanent fragmentation. Animals.

[ref-12] Bergh A, Lund I, Bostrom A, Hyytiainen H, Asplund K (2021). A systematic review of complementary and alternative veterinary medicine: miscellaneous therapies. Animals.

[ref-13] Bird NC, Abels JR, Richardson SS (2020). Histology and structural integration of the major morphologies of the Cypriniform Weberian apparatus. Journal of Morphology.

[ref-14] Bispham J (2006). Rhythm in music: what is it? Who has it? And why?. Music Perception.

[ref-15] Brooker JS (2016). An investigation of the auditory perception of western lowland gorillas in an enrichment study. Zoo Biology.

[ref-16] Brown S, Martinez MJ, Parsons LM (2004). Passive music listening spontaneously engages limbic and paralimbic systems. Neuroreport.

[ref-17] Bruce LL, Neary TJ (1995). The limbic system of tetrapods: a comparative analysis of cortical and amygdalar populations. Brain Behavior and Evolution.

[ref-18] Bryant GA (2013). Animal signals and emotion in music: coordinating affect across groups. Frontiers in Psychology.

[ref-19] Cainelli E, Vedovelli L, Bisiacchi P (2024). The mother–child interface: a neurobiological metamorphosis. Neuroscience.

[ref-20] Campo JL, Gil MG, Dávila SG (2005). Effects of specific noise and music stimuli on stress and fear levels of laying hens of several breeds. Applied Animal Behaviour Science.

[ref-21] Capshaw G, Christensen-Dalsgaard J, Carr CE (2022). Hearing without a tympanic ear. Journal of Experimental Biology.

[ref-22] Cartolano MC, Berenshtein I, Heuer RM, Pasparakis C, Rider M, Hammerschlag N, Paris CB, Grosell M, McDonald MD (2020). Impacts of a local music festival on fish stress hormone levels and the adjacent underwater soundscape. Environmental Pollution.

[ref-23] Catli T, Yildirim O, Turker A (2015). The effect of different tempos of music during feeding, on growth performance, chemical body composition, and feed utilization of turbot (*Psetta maeotica*, Pallas 1814). Israeli Journal of Aquaculture-Bamidgeh.

[ref-24] Celma-Miralles A, Toro JM (2020). Non-human animals detect the rhythmic structure of a familiar tune. Psychonomic Bulletin & Review.

[ref-25] Chanda ML, Levitin DJ (2013). The neurochemistry of music. Trends in Cognitive Sciences.

[ref-26] Chase AR (2001). Music discriminations by carp (*Cyprinus carpio*). Animal Learning and Behavior.

[ref-27] Chase ID (2006). Music notation: a new method for visualizing social interaction in animals and humans. Frontiers in Zoology.

[ref-28] Chatterjee D, Hegde S, Thaut M (2021). Neural plasticity: the substratum of music-based interventions in neurorehabilitation. NeuroRehabilitation.

[ref-29] Chen S, Liang T, Zhou FH, Cao Y, Wang C, Wang F-Y, Li F, Zhou X-F, Zhang J-Y, Li C-Q (2019). Regular music exposure in juvenile rats facilitates conditioned fear extinction and reduces anxiety after foot shock in adulthood. BioMed Research International.

[ref-30] Christensen CB, Christensen-Dalsgaard J, Madsen PT (2015). Hearing of the African lungfish (*Protopterus annectens*) suggests underwater pressure detection and rudimentary aerial hearing in early tetrapods. The Journal of Experimental Biology.

[ref-31] Christensen-Dalsgaard J, Brandt C, Wilson M, Wahlberg M, Madsen PT (2011). Hearing in the African lungfish (*Protopterus annectens*): pre-adaptation to pressure hearing in tetrapods?. Biology Letters.

[ref-32] Christensen-Dalsgaard J, Carr CE (2008). Evolution of a sensory novelty: tympanic ears and the associated neural processing. Brain Research Bulletin.

[ref-33] Clack JA (1989). Discovery of the earliest-known tetrapod stapes. Nature.

[ref-34] Constantin L, Poulsen RE, Scholz LA, Favre-Bulle IA, Taylor MA, Sun B, Goodhill GJ, Vanwalleghem GC, Scott EK (2020). Altered brain-wide auditory networks in a zebrafish model of fragile X syndrome. BMC Biology.

[ref-35] Contreras-Torres EG, Hernández-Chavez JF, Díaz-Quiroz CA, Molina-Barrios R, Millán PA, Ulloa-Mercado RG (2024). Music enrichment improves the behavior and leukocyte profile of dairy cattle. Open Agriculture.

[ref-36] Cyroń S, Czyz K, Patkowska-Sokoła B (2018). Effect of classical music on dog’s behavior in the shelter. Wiadomości Zootechniczne, R. LVI.

[ref-37] De Souza JF, Silveira MM, Barcellos HHA, Barcellos LJG, Luchiari AC (2022). Sound stimulus effects on dusky damselfish behavior and cognition. Marine Pollution Bulletin.

[ref-38] Dos Santos AC, De Abreu MS, De Mello GP, Costella V, Amaral NRdo, Zanella A, Poletto J, Petersen EV, Kalueff AV, Giacomini A (2023). Solfeggio-frequency music exposure reverses cognitive and endocrine deficits evoked by a 24-h light exposure in adult zebrafish. Behavioural Brain Research.

[ref-39] Dos Santos Lemes Lechuga KK, Caldara FR, De Castro Burbarelli MF, Odakura AM, dos Ouros CC, Garcia RG, Félix GA, De Lima Almeida Paz IC, Oliveira dos Santos VM, Braz JM (2023). Music and tactile stimuli during daily milking affect the welfare and productivity of dairy cows. Animals.

[ref-40] Eagan BH (2008). The effect of animal shelter sound on cat behaviour and welfare.

[ref-41] Engler WJ, Bain M (2017). Effect of different types of classical music played at a veterinary hospital on dog behavior and owner satisfaction. Journal of the American Veterinary Medical Association.

[ref-42] Erbe C, Dent ML, Gannon WL, McCauley RD, Römer H, Southall BL, Stansbury AL, Stoeger AS, Thomas JA (2022). The effects of noise on animals. Exploring Animal Behavior Through Sound.

[ref-43] Ettenberger M, Bieleninik L, Epstein S, Elefant C (2021). Defining attachment and bonding: overlaps, differences and implications for music therapy clinical practice and research in the neonatal intensive care unit (NICU). International Journal of Environmental Research and Public Health.

[ref-44] Evans HM (1930). The swim-bladder and weberian ossicles and their relation to hearing in fishes. Proceedings of the Royal Society of Medicine.

[ref-45] Fan Y, Fang K, Sun R, Shen D, Yang J, Tang Y, Fang G (2022). Hierarchical auditory perception for species discrimination and individual recognition in the music frog. Current Zoology.

[ref-46] Fanning L, Larsen H, Taylor PS (2020). A preliminary study investigating the impact of musical concerts on the behavior of captive fiordland penguins (Eudyptes pachyrhynchus) and collared peccaries (Pecari tajacu). Animals.

[ref-47] Ferreri L, Mas-Herrero E, Zatorre RJ, Ripolles P, Gomez-Andres A, Alicart H, Olive G, Marco-Pallares J, Antonijoan RM, Valle M, Riba J, Rodriguez-Fornells A (2019). Dopamine modulates the reward experiences elicited by music. Proceedings of the National Academy of Sciences of the United States of America.

[ref-48] Fettiplace R (2020). Diverse mechanisms of sound frequency discrimination in the vertebrate cochlea. Trends in Neurosciences.

[ref-49] Filippi P, Hoeschele M, Spierings M, Bowling DL (2019). Temporal modulation in speech, music, and animal vocal communication: evidence of conserved function. Annals of the New York Academy of Sciences.

[ref-50] Fitch WT (2006). The biology and evolution of music: a comparative perspective. Cognition.

[ref-51] Flores-García M, Flores Á, Aso E, Otero-López P, Ciruela F, Videla S, Grau-Sánchez J, Rodríguez-Fornells A, Bonaventura J, Fernández-Dueñas V (2025). Dopamine dynamics in chronic pain: music-induced, sex-dependent, behavioral effects in mice. Pain Reports.

[ref-52] Fritzsch B, Elliott KL (2023). Fish hearing revealed: do we understand hearing in critical fishes and marine tetrapods. Journal of the Acoustical Society of America.

[ref-53] Fritzsch B, Schultze HP, Elliott KL (2023). The evolution of the various structures required for hearing in Latimeria and tetrapods. IBRO Neuroscience Reports.

[ref-54] Fritzsch B, Straka H (2014). Evolution of vertebrate mechanosensory hair cells and inner ears: toward identifying stimuli that select mutation driven altered morphologies. The Journal of Comparative Physiology A: Neuroethology, Sensory, Neural, and Behavioral Physiology.

[ref-55] Gao X, Gong J, Yang B, Liu Y, Xu H, Hao Y, Jing J, Feng Z, Li L (2023). Effect of classical music on growth performance, stress level, antioxidant index, immune function and meat quality in broilers at different stocking densities. Frontiers in Veterinary Science.

[ref-56] Gebhart V, Buchberger W, Klotz I, Neururer S, Rungg C, Tucek G, Zenzmaier C, Perkhofer S (2020). Distraction-focused interventions on examination stress in nursing students: effects on psychological stress and biomarker levels. A randomized controlled trial. International Journal of Nursing Practice.

[ref-57] Georgiou SG, Anagnostou TL, Sideri AI, Gouletsou PG, Athanasiou LV, Kazakos G, Tsioli V, Dermisiadou E, Galatos AD (2024). Effect of classical music on light-plane anaesthesia and analgesia in dogs subjected to surgical nociceptive stimuli. Scientific Reports.

[ref-58] Ginovart-Panisello GJ, Alsina-Pages RM, Sanz II, Monjo TP, Prat MC (2020). Acoustic description of the soundscape of a real-life intensive farm and its impact on animal welfare: a preliminary analysis of farm sounds and bird vocalisations. Sensors.

[ref-59] González A, Northcutt RG (2009). An immunohistochemical approach to lungfish telencephalic organization. Brain Behavior and Evolution.

[ref-60] Gray PM, Krause B, Atema J, Payne R, Krumhansl C, Baptista L (2001). Biology and music. The music of nature. Science.

[ref-61] Greenberg DM, Decety J, Gordon I (2021). The social neuroscience of music: understanding the social brain through human song. American Psychologist.

[ref-62] Guérineau C, Lõoke M, Ganassin G, Bertotto D, Bortoletti M, Cavicchioli L, Furlati S, Mongillo P, Marinelli L (2022). Enrichment with classical music enhances affiliative behaviours in bottlenose dolphin. Applied Animal Behaviour Science.

[ref-63] Guinot G, Cavin L (2016). ‘Fish’ (Actinopterygii and Elasmobranchii) diversification patterns through deep time. Biological Reviews of the Cambridge Philosophical Society.

[ref-64] Hampton A, Ford A, Cox RE, Liu CC, Koh R (2020). Effects of music on behavior and physiological stress response of domestic cats in a veterinary clinic. Journal of Feline Medicine and Surgery.

[ref-65] Harada Y, Kasuga S, Tamura S (2001). Comparison and evolution of the lagena in various animal species. Acta Oto-Laryngologica.

[ref-66] Harley JJ, Rowden LJ, Clifforde LM, Power A, Stanley CR (2022). Preliminary investigation of the effects of a concert on the behavior of zoo animals. Zoo Biology.

[ref-67] Hart NS, Collin SP (2015). Sharks senses and shark repellents. Integrative Zoology.

[ref-68] Hasani H, Sun J, Zhu SI, Rong Q, Willomitzer F, Amor R, McConnell G, Cossairt O, Goodhill GJ (2023). Whole-brain imaging of freely-moving zebrafish.

[ref-69] Hayes ME, Hemsworth LM, Morrison RS, Butler KL, Rice M, Rault JL, Hemsworth PH (2021). Effects of positive human contact during gestation on the behaviour, physiology and reproductive performance of sows. Animals.

[ref-70] Hewitt M (2008). Musical acoustics: an introduction.

[ref-71] Howe K, Clark MD, Torroja CF, Torrance J, Berthelot C, Muffato M, Collins JE, Humphray S, McLaren K, Matthews L, McLaren S, Sealy I, Caccamo M, Churcher C, Scott C, Barrett JC, Koch R, Rauch GJ, White S, Chow W, Kilian B, Quintais LT, Guerra-Assunção JA, Zhou Y, Gu Y, Yen J, Vogel JH, Eyre T, Redmond S, Banerjee R, Chi J, Fu B, Langley E, Maguire SF, Laird GK, Lloyd D, Kenyon E, Donaldson S, Sehra H, Almeida-King J, Loveland J, Trevanion S, Jones M, Quail M, Willey D, Hunt A, Burton J, Sims S, McLay K, Plumb B, Davis J, Clee C, Oliver K, Clark R, Riddle C, Elliot D, Threadgold G, Harden G, Ware D, Begum S, Mortimore B, Kerry G, Heath P, Phillimore B, Tracey A, Corby N, Dunn M, Johnson C, Wood J, Clark S, Pelan S, Griffiths G, Smith M, Glithero R, Howden P, Barker N, Lloyd C, Stevens C, Harley J, Holt K, Panagiotidis G, Lovell J, Beasley H, Henderson C, Gordon D, Auger K, Wright D, Collins J, Raisen C, Dyer L, Leung K, Robertson L, Ambridge K, Leongamornlert D, McGuire S, Gilderthorp R, Griffiths C, Manthravadi D, Nichol S, Barker G, Whitehead S, Kay M, Brown J, Murnane C, Gray E, Humphries M, Sycamore N, Barker D, Saunders D, Wallis J, Babbage A, Hammond S, Mashreghi-Mohammadi M, Barr L, Martin S, Wray P, Ellington A, Matthews N, Ellwood M, Woodmansey R, Clark G, Cooper J, Tromans A, Grafham D, Skuce C, Pandian R, Andrews R, Harrison E, Kimberley A, Garnett J, Fosker N, Hall R, Garner P, Kelly D, Bird C, Palmer S, Gehring I, Berger A, Dooley CM, Ersan-Ürün Z, Eser C, Geiger H, Geisler M, Karotki L, Kirn A, Konantz J, Konantz M, Oberländer M, Rudolph-Geiger S, Teucke M, Lanz C, Raddatz G, Osoegawa K, Zhu B, Rapp A, Widaa S, Langford C, Yang F, Schuster SC, Carter NP, Harrow J, Ning Z, Herrero J, Searle SM, Enright A, Geisler R, Plasterk RH, Lee C, Westerfield M, De Jong PJ, Zon LI, Postlethwait JH, Nüsslein-Volhard C, Hubbard TJ, Crollius HRoest, Rogers J, Stemple DL (2013). The zebrafish reference genome sequence and its relationship to the human genome. Nature.

[ref-72] Huo X, Wongkwanklom M, Phonraksa T, Na-Lampang P (2021). Effects of playing classical music on behavior of stabled horses. Veterinary Integrative Sciences.

[ref-73] Imanpoor MR, Gholampour TE, Zolfaghari M (2011). Effect of light and music on growth performance and survival rate of goldfish (*Carassius auratus*). Iranian Journal of Fisheries Sciences.

[ref-74] Jacob FG, Nääs IdA, Salgado DDA, Baracho MdS, Lima NDdS, Pereira DF (2022). Does environmental enrichment with music and strobe light affect broilers’ welfare? analyzing their on-farm reaction. AgriEngineering.

[ref-75] Johnson A, Loh E, Verbitsky R, Slessor J, Franczak BC, Schalomon M, Hamilton TJ (2023). Examining behavioural test sensitivity and locomotor proxies of anxiety-like behaviour in zebrafish. Scientific Reports.

[ref-76] Jung DH, Kim NY, Moon SH, Jhin C, Kim HJ, Yang JS, Kim HS, Lee TS, Lee JY, Park SH (2021). Deep learning-based cattle vocal classification model and real-time livestock monitoring system with noise filtering. Animals.

[ref-77] Kamar N, Md Yusof NN (2023). The impact of music on milk production and behaviour of dairy cattle. Pertanika Journal of Tropical Agricultural Science.

[ref-78] Kemp A (2020). The effects of music on dairy production. Honors College Theses.

[ref-79] Kinsler LE, Frey AR, Coppens AB, Sanders JV (2000). Fundamentals of Acoustics.

[ref-80] Kochewad SA, Gaur GK, Maurya VP, Bharti PK, Sahoo NR, Pandey HO, Singh M, Verma MR (2021). Effect of milking environment enrichment through music on production performance and behaviour in cattle. Tropical Animal Health and Production.

[ref-81] Korsós G, Horváth K, Lukács A, Vezér T, Glávits R, Fodor K, Fekete SG (2018). Effects of accelerated human music on learning and memory performance of rats. Applied Animal Behaviour Science.

[ref-82] Kühlmann AYR, De Rooij A, Hunink MGM, De Zeeuw CI, Jeekel J (2018). Music affects rodents: a systematic review of experimental research. Frontiers in Behavioral Neuroscience.

[ref-83] Kumar S, Suleski M, Craig JM, Kasprowicz AE, Sanderford M, Li M, Stecher G, Hedges SB (2022). TimeTree 5: an expanded resource for species divergence times. Molecular Biology and Evolution.

[ref-84] Ladich F (2000). Acoustic communication and the evolution of hearing in fishes. Philosophical Transactions of the Royal Society B.

[ref-85] Ladich F (2014). Fish bioacoustics. Current Opinion in Neurobiology.

[ref-86] Lara RA, Vasconcelos RO (2021). Impact of noise on development, physiological stress and behavioural patterns in larval zebrafish. Scientific Reports.

[ref-87] Laurijs KA, Briefer EF, Reimert I, Webb LE (2021). Vocalisations in farm animals: a step towards positive welfare assessment. Applied Animal Behaviour Science.

[ref-88] Lee V, Pawlisch B, Macedo-Lima M, Remage-Healey L (2017). Norepinephrine enhances song responsiveness and encoding in the auditory forebrain of male zebra finches. Journal of Neurophysiology.

[ref-89] Li J, Li X, Liu H, Li J, Han Q, Wang C, Zeng X, Li Y, Ji W, Zhang R, Bao J (2021a). Effects of music stimulus on behavior response, cortisol level, and horizontal immunity of growing pigs. Journal of Animal Science.

[ref-90] Li Y, Jia Z, Zhang S, He X (2021b). Progress in gene-editing technology of zebrafish. Biomolecules.

[ref-91] Li X, Zhao JN, Zhao P, Zhang X, Bi YJ, Li JH, Liu HG, Wang C, Bao J (2019). Behavioural responses of piglets to different types of music. Animal.

[ref-92] Liang D, Shen XX, Zhang P (2013). One thousand two hundred ninety nuclear genes from a genome-wide survey support lungfishes as the sister group of tetrapods. Molecular Biology and Evolution.

[ref-93] Lidicker W (2019). Music and dance in mammals. Therya.

[ref-94] Lieber AC, Bose J, Zhang X, Seltzberg H, Loewy J, Rossetti A, Mocco J, Kellner CP (2019). Effects of music therapy on anxiety and physiologic parameters in angiography: a systematic review and meta-analysis. Journal of NeuroInterventional Surgery.

[ref-95] Lindig AM, McGreevy PD, Crean AJ (2020). Musical dogs: a review of the influence of auditory enrichment on canine health and behavior. Animals.

[ref-96] Lipovsek M, Elgoyhen AB (2023). The evolutionary tuning of hearing. Trends in Neurosciences.

[ref-97] Lippi ICC, Caldara FR, Almeida-Paz ICL, Morais HB, Odakura AM, Konkiewitz EC, Ferreira WS, Fraga TL, Burbarelli MFC, Felix GA, Garcia RG, Santos LSD (2022). Effects of music therapy on neuroplasticity, welfare, and performance of piglets exposed to music therapy in the intra- and extra-uterine phases. Animals.

[ref-98] Long JA, Gordon MS (2004). The greatest step in vertebrate history: a paleobiological review of the fish-tetrapod transition. Physiological and Biochemical Zoology.

[ref-99] Lukasz D, Kindt KS (2018). *In vivo* calcium imaging of lateral-line hair cells in larval zebrafish. Journal of Visualized Experiments.

[ref-100] Marchetto L, Barcellos LJG, Koakoski G, Soares SM, Pompermaier A, Maffi VC, Costa R, Silva CGda, Zorzi NR, Demin KA, Kalueff AV, Barcellos HHDe Alcantara (2021). Auditory environmental enrichment prevents anxiety-like behavior, but not cortisol responses, evoked by 24-h social isolation in zebrafish. Behavioural Brain Research.

[ref-101] Monesson-Olson BD, Browning-Kamins J, Aziz-Bose R, Kreines F, Trapani JG (2014). Optical stimulation of zebrafish hair cells expressing channelrhodopsin-2. PLOS ONE.

[ref-102] Moore BCJ (2012). An introduction to the psychology of hearing.

[ref-103] Nikolsky A (2020). The pastoral origin of semiotically functional tonal organization of music. Frontiers in Psychology.

[ref-104] Niu TY, Xiang BC, Huang PY, Yang XG, Chai LH (2025). Effects of music enrichment on the welfare of small ornamental fishes. Aquaculture Reports.

[ref-105] Orihuela A, Mota-Rojas D, Strappini A, Serrapica F, Braghieri A, Mora-Medina P, Napolitano F (2021). Neurophysiological mechanisms of mother-young bonding in buffalo and other farm animals. Animals.

[ref-106] Panksepp J (1986). The neurochemistry of behavior. Annual Review of Psychology.

[ref-107] Papoutsoglou SE, Karakatsouli N, Batzina A, Papoutsoglou ES, Tsopelakos A (2008). Effect of music stimulus on gilthead seabream Sparus aurata physiology under different light intensity in a re-circulating water system. Journal of Fish Biology.

[ref-108] Papoutsoglou SE, Karakatsouli N, Louizos E, Chadio S, Kalogiannis D, Dalla C, Polissidis A, Papadopoulou-Daifoti Z (2007). Effect of Mozart’s music (Romanze-Andante of Eine Kleine Nacht Musik, sol major, K525) stimulus on common carp (*Cyprinus carpio* L.) physiology under different light conditions. Aquacultural Engineering.

[ref-109] Papoutsoglou SE, Karakatsouli N, Papoutsoglou ES, Vasilikos G (2010). Common carp (*Cyprinus carpio*) response to two pieces of music (Eine Kleine Nachtmusik and Romanza) combined with light intensity, using recirculating water system. Fish Physiology and Biochemistry.

[ref-110] Papoutsoglou SE, Karakatsouli N, Psarrou A, Apostolidou S, Papoutsoglou ES, Batzina A, Leondaritis G, Sakellaridis N (2015). Gilthead seabream (Sparus aurata) response to three music stimuli (Mozart-Eine Kleine Nachtmusik, Anonymous-Romanza, Bach-Violin Concerto No. 1) and white noise under recirculating water conditions. Fish Physiology and Biochemistry.

[ref-111] Papoutsoglou SE, Karakatsouli N, Skouradakis C, Papoutsoglou ES, Batzina A, Leondaritis G, Sakellaridis N (2013). Effect of musical stimuli and white noise on rainbow trout (*Oncorhynchus mykiss*) growth and physiology in recirculating water conditions. Aquacultural Engineering.

[ref-112] Paszkiel S, Dobrakowski P, Lysiak A (2020). The impact of different sounds on stress level in the context of EEG, cardiac measures and subjective stress level: a pilot study. Brain Sciences.

[ref-113] Pfaff C, Schultz JA, Schellhorn R (2019). The vertebrate middle and inner ear: A short overview. Journal of Morphology.

[ref-114] Piitulainen R, Hirskyj-Douglas I (2020). Music for monkeys: building methods to design with white-faced sakis for animal-driven audio enrichment devices. Animals.

[ref-115] Plack CJ (2023). The sense of hearing.

[ref-116] Poćwierz-Marciniak I, Harciarek M (2021). The effect of musical stimulation and mother’s voice on the early development of musical abilities: a neuropsychological perspective. International Journal of Environmental Research and Public Health.

[ref-117] Poppelier T, Bonsberger J, Berkhout BW, Pollmanns R, Schluessel V (2022). Acoustic discrimination in the grey bamboo shark Chiloscyllium griseum. Scientific Reports.

[ref-118] Popper AN, Hawkins AD, Sisneros JA (2022). Fish hearing specialization—a re-evaluation. Hearing Research.

[ref-119] Putland RL, Montgomery JC, Radford CA (2019). Ecology of fish hearing. Journal of Fish Biology.

[ref-120] Riemer S, Heritier C, Windschnurer I, Pratsch L, Arhant C, Affenzeller N (2021). A review on mitigating fear and aggression in dogs and cats in a veterinary setting. Animals.

[ref-121] Rosen DE, Greenwood PH, Anderson S, Weitzman SH (1970). Origin of the Weberian apparatus and the relationships of the ostariophysan and gonorynchiform fishes. American Museum Novitates.

[ref-122] Schartl M, Woltering JM, Irisarri I, Du K, Kneitz S, Pippel M, Brown T, Franchini P, Li J, Li M, Adolfi M, Winkler S, Sousa JDe Freitas, Chen Z, Jacinto S, Kvon EZ, De Oliveira LRCorrea, Monteiro E, Amaral DBaia, Burmester T, Chalopin D, Suh A, Myers E, Simakov O, Schneider I, Meyer A (2024). The genomes of all lungfish inform on genome expansion and tetrapod evolution. Nature.

[ref-123] Shinozuka K, Ono H, Watanabe S (2013). Reinforcing and discriminative stimulus properties of music in goldfish. Behav Processes.

[ref-124] Silva FRS, Kods Miranda, Smds Piedade, Salgado DDA (2017). Effect of auditory enrichment (music) in pregnant sows welfare. Engenharia Agrícola.

[ref-125] Singh N, Sharma D (2022). A review study of impact assessment of musical sounds in the context of physical, behavioural and dietary aspects of fishes. Journal of Experimental Zoology India.

[ref-126] Snowdon CT (2021). Animal signals, music and emotional well-being. Animals.

[ref-127] Stomberg I, Tiderman-Österberg J (2021). Duets with nature: how natural acoustics affect the experience of performing Nordic herding music in outdoor settings. AAWM Music and Nature.

[ref-128] Sugihara T, Diltz MD, Averbeck BB, Romanski LM (2006). Integration of auditory and visual communication information in the primate ventrolateral prefrontal cortex. Journal of Neuroscience.

[ref-129] Takechi M, Kitazawa T, Hirasawa T, Hirai T, Iseki S, Kurihara H, Kuratani S (2016). Developmental mechanisms of the tympanic membrane in mammals and non-mammalian amniotes. Congenit Anom (Kyoto).

[ref-130] Toader C, Tataru CP, Florian IA, Covache-Busuioc RA, Bratu BG, Glavan LA, Bordeianu A, Dumitrascu DI, Ciurea AV (2023). Cognitive crescendo: how music shapes the brain’s structure and function. Brain Sciences.

[ref-131] Toth B, Bársony P, Kusza S (2025). Noise sources and music stimuli in teleost fish aquaculture systems-a review. Fishes.

[ref-132] Vila Pouca C, Brown C (2018). Food approach conditioning and discrimination learning using sound cues in benthic sharks. Animal Cognition.

[ref-133] Wang YL, Lin CY, Huang SP, Lee CY, Tuanmu MN, Wang TY (2022). Chub movement is attracted by the collision sounds associated with spawning activities. Zootaxa.

[ref-134] Wang K, Wang J, Zhu C, Yang L, Ren Y, Ruan J, Fan G, Hu J, Xu W, Bi X, Zhu Y, Song Y, Chen H, Ma T, Zhao R, Jiang H, Zhang B, Feng C, Yuan Y, Gan X, Li Y, Zeng H, Liu Q, Zhang Y, Shao F, Hao S, Zhang H, Xu X, Liu X, Wang D, Zhu M, Zhang G, Zhao W, Qiu Q, He S, Wang W (2021). African lungfish genome sheds light on the vertebrate water-to-land transition. Cell.

[ref-135] Wells DL, Irwin RM (2008). Auditory stimulation as enrichment for zoo-housed Asian elephants (*Elephas maximus*). Animal Welfare.

[ref-136] Wong MI, Lau IH, Gordillo-Martinez F, Vasconcelos RO (2022). The effect of time regime in noise exposure on the auditory system and behavioural stress in the zebrafish. Scientific Reports.

[ref-137] Xiao Y, Rong D, Ye H, Duan Y, Qiao L, Zuo L, Liu L, Bayram H, Wang J (2025). Music aggravates catechol-induced behavioural abnormality and redox imbalance in Zebrafish. International Journal of Developmental Neuroscience.

[ref-138] Yang CX, Zhao J, Xu MM, Ji BT, Li JX, Wang JX, Yang XY (2025). Evaluation of the impact of music on antioxidant mechanisms and survival in salt-stressed goldfish. Open Chemistry.

[ref-139] Zapata-Cardona J, Ceballos MC, Rodriguez BJ (2024). Music and emotions in non-human animals from biological and comparative perspectives. Animals.

[ref-140] Zhang R, Liu Q, Pan S, Zhang Y, Qin Y, Du X, Yuan Z, Lu Y, Song Y, Zhang M, Zhang N, Ma J, Zhang Z, Jia X, Wang K, He S, Liu S, Ni M, Liu X, Xu X, Yang H, Wang J, Seim I, Fan G (2023). A single-cell atlas of West African lungfish respiratory system reveals evolutionary adaptations to terrestrialization. Nature Communications.

